# Regulation of Interferon-Stimulated Gene BST2 by a lncRNA Transcribed from a Shared Bidirectional Promoter

**DOI:** 10.3389/fimmu.2014.00676

**Published:** 2015-01-30

**Authors:** Hiroto Kambara, Lalith Gunawardane, Elizabeth Zebrowski, Lenche Kostadinova, Raul Jobava, Dawid Krokowski, Maria Hatzoglou, Donald D. Anthony, Saba Valadkhan

**Affiliations:** ^1^Department of Biochemistry, Case Western Reserve University School of Medicine, Cleveland, OH, USA; ^2^Divisions of Infectious and Rheumatic Diseases, Department of Medicine, Case Western Reserve University School of Medicine, Cleveland, OH, USA; ^3^Department of Nutrition, Case Western Reserve University School of Medicine, Cleveland, OH, USA

**Keywords:** lncRNAs, bidirectional promoters, BST2, transcriptional regulation, IFN response

## Abstract

Recent genome-wide studies have revealed the presence of thousands of long non-protein-coding RNAs (lncRNAs), some of which may play critical roles in the cell. We have previously shown that a large number of lncRNAs show differential expression in response to interferon (IFN)α stimulation in primary human cells. Here, we show that a subset of IFN-induced lncRNAs are positioned in proximity of protein-coding IFN-stimulated genes (ISGs). The majority of gene pairs originated from bidirectional promoters and showed positively correlated expression. We focused our analysis on a pair consisting of the known protein-coding ISG, BST2, and an un-studied putative lncRNA originating from the promoter region of BST2 in a divergent orientation. We showed that this transcript was a multi-exonic, polyadenylated long RNA that lacked protein-coding capacity. BST2 and the lncRNA were both induced in response to IFNα in diverse cell types. The induction of both genes was mediated through the JAK–STAT pathway, suggesting that IFN-stimulated response elements within the shared promoter activated the transcription of both genes. RNAi-mediated knock-down of the lncRNA resulted in down-regulation of BST2, and we could show that this down-regulation occurred at the level of transcription. Forced overexpression of this lncRNA, which we named BST2 IFN-Stimulated Positive Regulator (BISPR), resulted in up-regulation of BST2, indicating that the regulation of expression of BST2 by BISPR is mediated through interactions involving BISPR RNA itself, rather than the impact of its transcription from an adjacent locus. Importantly, upon IFN stimulation, transcriptional activation of BISPR preceded the induction of BST2, suggesting that expression of BISPR facilitated the initiation of transcription in its paired protein-coding gene. The lncRNA-mediated transcriptional regulation described in this study may help govern the expression of additional protein-coding RNAs involved in IFN response and other cellular processes.

## Introduction

The interferon (IFN) response is a critical arm of innate immunity and constitutes a major host defense mechanism against invasion of pathogens such as viruses and bacteria (1–3). Release of IFNs, which mainly occurs in response to viral and microbial encounter, results in signaling through the IFN receptors, which in turn leads to activation of the JAK–STAT pathway (2–4). The final outcome of this signaling pathway is the formation of a transcriptional activator complex between phosphorylated STAT1, STAT2, and the transcription factor Interferon Regulatory Factor 9 (IRF9). The STAT1/STAT2/IRF9 ternary complex binds to the IFN-stimulated response elements (ISREs) found in promoter of IFN-responsive genes, resulting in up-regulation of expression of a large number of host genes. While the function of many IFN-stimulated genes (ISGs) remains un-studied, analysis of a subset of them has indicated their involvement in various aspects of antiviral defense and immune modulatory functions (1–4).

Recent large scale transcriptome analyses have revealed the existence of tens of thousands of long non-protein-coding RNAs (lncRNAs), which play crucial roles in diverse aspects of cellular function including several steps in both innate and adaptive immune responses (5–14). However, the role of this class of transcripts in the IFN response had not been investigated. Recently, using a high-throughput RNA sequencing approach on IFN-stimulated primary human cells, we provided evidence for the presence of tens of lncRNAs which showed a robust transcriptional up-regulation in response to IFN (15). Analysis of the function of one of the upregulated lncRNAs, Negative Regulator of the IFN Response (NRIR, originally named lncRNA-CMPK2), proved that it was a negative regulator of the IFN response and functioned as a transcriptional repressor of a subset of protein-coding ISGs (15). While the function of all the other identified IFN-induced lncRNAs remain unknown, it is likely that similar to NRIR, many of them perform important roles as regulators or effector molecules in the IFN response.

Current studies of the function of lncRNAs indicate that many of them regulate the expression of other genes through diverse mechanisms including induction of changes in the structure of chromatin and recruitment of various effector molecules such as transcriptional complexes, or direct interactions with other RNAs such as miRNAs or protein-coding RNAs (6, 7, 16–19). In many cases, lncRNAs seem to regulate the expression of protein-coding or other lncRNA genes which are located in their vicinity and thus, analysis of the genomic locus of lncRNAs and their relative positioning compared to the nearby genes can provide important clues into their function (7, 16, 20). For example, overlap of the locus of a lncRNA with the promoter region of another gene can result in interference with the formation of transcriptional complexes on the promoter of the overlapped gene by run-through transcription, or alternatively make it accessible to transcriptional complexes. Even in the absence of overlap, a nearby lncRNA gene can affect the expression of nearby genes in a positive or negative manner, for example by binding and increasing the local concentration of effector molecules such as chromatin modifying factors, resulting in local modulation of chromatin structure. The impact of lncRNA transcription on expression of protein-coding genes is particularly important as it has been shown that promoters are inherently bidirectional, and a large fraction of human protein-coding genes have a non-coding transcript originating from their promoters in the opposite direction (21–25). While the majority of such promoter-upstream transcription results in short, unstable RNAs, in a subset of genes it results in stable, longer RNAs that in many cases are spliced and polyadenylated (26–28). The presence of such “bidirectional” promoters generating coordinately expressed pairs of protein-coding genes has been known for over a decade (21, 22, 29). In many cases, the paired genes perform related functions and thus, their transcription from the same promoter facilitates their coordinated expression (22, 29). More recent discovery of the origination of protein-coding/lncRNA pairs from bidirectional promoters has raised the intriguing possibility that the pairs may similarly perform related functions or that the lncRNAs may regulate the expression of their paired protein-coding genes through the mechanisms described above and elsewhere in literature (5, 25, 27). While the global analysis of protein-coding/lncRNA pairs originating from bidirectional promoters has indicated that the majority of them show coordinated expression, some pairs are expressed in a mutually exclusive fashion, suggesting that a diversity of regulatory mechanisms can be at work in such loci (23, 25, 27, 30). Functional study of a small number of these promoters has confirmed this point, as knock down experiments on one of the two transcripts in such pairs has resulted in both up- and down-regulation of the other member of the pair, suggesting that such paired RNAs may engage in complex regulatory interactions (30–32). Considering the abundance of protein-coding/lncRNA pairs originating from bidirectional promoters, it is likely that these regulatory interactions play a prominent role in the overall control of gene expression.

As a first step toward understanding the role of protein-coding/lncRNA pairs in regulation of the IFN response, we identified lncRNA and protein-coding RNAs which were located in proximity of each other and showed differential expression in response to IFN stimulation. All identified pairs showed a correlated transcriptional response to IFN, and included a number of known protein-coding ISGs. Functional analysis of one such loci indicated the presence of a stable, spliced, and polyadenylated long non-coding RNA resulting from apparent bidirectional transcription from the BST2 (Tetherin) promoter. The expression of the lncRNA in response to IFN was mediated through the JAK–STAT pathway and correlated with the expression of BST2 in diverse cell types. Interestingly, forced overexpression and knock down of the lncRNA resulted in an increase and decrease in expression of BST2, respectively, suggesting that the expression of lncRNA, which we name BST2 IFN-stimulated positive regulator (BISPR), was needed for efficient transcription of its paired protein-coding gene. We could show that this regulation was at the level of transcription, and importantly, that upon IFN stimulation, the rise in the level of BISPR preceded the induction of BST2, suggesting that the transcription of BISPR facilitated the initiation of transcription in its paired protein-coding gene. Intriguingly, the putative bidirectional promoter giving rise to BISPR and BST2 overlaps an enhancer, raising the possibility that both RNAs may have evolved from enhancer-associated (eRNA) transcripts.

## Materials and Methods

### Computational analyses

The paired-end 100 bp-long RNA-seq reads that formed the basis for this study originated from high-throughput transcriptome analysis of human primary hepatocytes obtained from five human donors (SRA accession number SRP045406) (15). Data were processed and analyzed as described (15). Protein-coding and non-coding genes that showed robust expression and significant IFN-mediated differential expression of over twofold in either direction were filtered for being in proximity of each other (defined by the maximum distance of <2000 bp between the two transcriptional units). The naming of novel transcripts was according to HGNC guidelines (33). Defining the open reading frames (ORFs) was performed as described (15). Statistical studies for determining the Spearman’s correlation coefficient in Figure 5E was performed as previously described (34).

### Cell culture experiments

Cell lines used in this study were kind gifts of Drs. Karn, Rice, and Dowlati (15). Primary human keratinocytes were obtained from neonatal foreskin and were passaged twice before being studied. For primary keratinocytes and natural killer (NK) and peripheral blood mononuclear cells (PBMC), subjects provided written informed consent for the use of their blood (for NK and PBMC cells) or foreskin (for primary keratinocytes) cells. The samples were obtained according to the guidelines of the 1975 Declaration of Helsinki with prior approval of the institutional review board for human studies at University Hospitals of Cleveland. The harvest of primary NK and PBMC cells from healthy donors was performed as previously described (35). The RNA from cells derived from individual donors was extracted and analyzed by RT-qPCR as described below. All cells were cultured at 37°C in a humidified atmosphere at 5% CO_2_ as described previously (15). Primary keratinocytes were cultured in keratinocyte-specific serum-free medium (Gibco 2015-16) supplemented by 5 ng/ml human recombinant EGF and 50 μg/ml bovine pituitary extract (Gibco 2015-10). Treatment of cells with IFNα, ruxolitinib, actinomycin D, IFNγ, and TNFα, and extraction of total cellular RNA was performed as described (15). Cellular fractionation into nuclear and cytoplasmic fractions was performed as previously described (34). 5′ and 3′ RACE (rapid amplification of cDNA ends) was performed using SMARTer RACE cDNA amplification kit from Clontech, as described (15).

### Polysome profile analysis

To investigate the association of BISPR with the translational machinery, we used human corneal epithelial cells, which showed a robust expression of BISPR and BST2 and allowed for unambiguous identification and quantitation of the RNAs in different polysome fractions. The 10.014 pRSVT cells (ATCC^®^ CRL-11515™) were incubated with 100 μg/ml cycloheximide for 5 min before harvesting. Cells were lysed in polysome lysis buffer (10 mM HEPES-KOH, pH 7.4, 7.5 mM MgCl2, 100 mM KCl, 1 mM dithiothreitol, 0.25% Non-idet P-40, 200 units/ml RNase inhibitor, 100 μg/ml cycloheximide, and one tablet of EDTA-free protease inhibitor mixture/10 ml (Roche Applied Science)] and homogenized by passing the lysate through a 23G needle 15 times. Lysates were centrifuged at 17,000 × *g* for 10 min; the supernatants were collected, and absorbance was measured. About seven A254 units of the cytosolic extracts were layered over 10–50% sucrose gradients and centrifuged at 17,000 rpm in a Beckman SW28 rotor for 15 h at 4°C. After centrifugation, fractions (~1.2 ml each) were collected. RNA was extracted with TRIzol reagent (Invitrogen) and used to determine the distribution of ribosomal RNAs (by agarose gel electrophoresis) and BISPR, BST2, and GAPDH transcripts (by RT-qPCR).

### RT-PCR

Preparation of cDNA was performed as described, using both oligo (dT) and random hexamers (15). For strand-specific RT-qPCR, the indicated reverse transcription primers were used in the RT reaction, followed by inactivation of the RT enzyme. The resulting cDNA was used in qPCR reactions with Biorad SYBR Green Kit (Biorad) on a Mastercycler Realplex2 system (Eppendorf) and analyzed as described (15). Error bars represent the SEM from at least two technical repeats and two biological repeats per experiment. To determine the statistical significance of observed differences, *p*-values were calculated using Student’s *t*-test with *p*-values <0.05 considered significant.

### Gene silencing

Knock-down of STAT2 was performed as previously described (15). Dicer-substrate interference RNAs (DsiRNAs) targeting BISPR and negative control DsiRNAs were purchased from Integrated DNA Technologies (DsiRNA#1 sense: GACACACAGAGUAUCCUUAACCCAC, antisense: GUGGGUUAAGGAUACUCUGUGUGUCUU, which target nucleotides 670–694 of BISPR close to the 5′ end of the last exon; DsiRNA#2 sense: CACUUAGGCAGGAGGAUCACUCGAG, antisense: CUCGAGUGAUCCUCCUGCCUAAGUGUU, which target nucleotides 546–570 of BISPR close to the 3′ end of the third exon). THP1 cells were seeded onto six-well plates and transfected with 20 nM final concentration of DsiRNAs using Lipofectamine ^®^ RNAiMax Reagent (Invitrogen) according to the manufacturer’s protocol. The transfected cells were incubated for 24 h in RPMI 1640 supplemented with 10% FBS and used for the downstream experiments. EZH2 knock down studies were performed on THP1 cells using shRNA constructs TRCN0000040074 and TRCN0000040077 targeting EZH2 and the non-targeting SHC002 shRNA construct from Sigma. Lentiviral preparation, cell transfection, and RNA harvest was performed as described (15).

## Results

**Figure 1:**
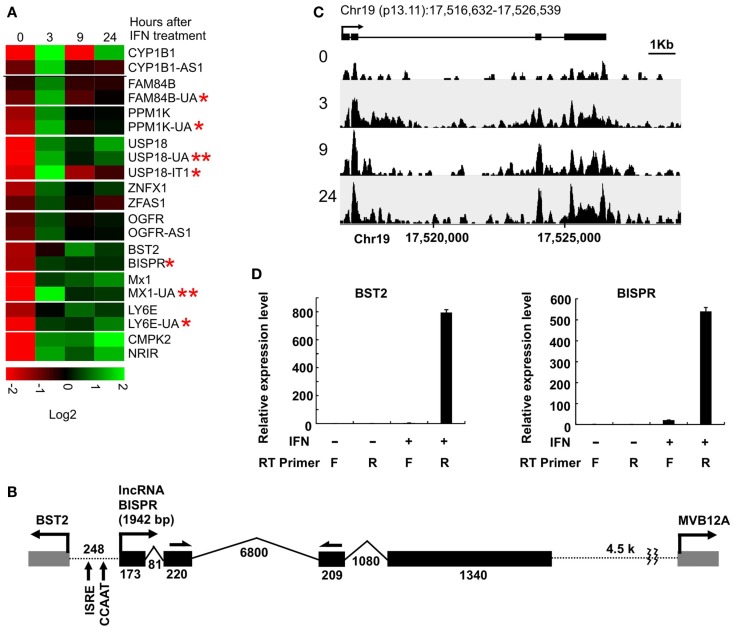
**Interferon-induced lncRNA/protein-coding RNA pairs show coordinated expression**. **(A)** Coordinated expression pattern of ten lncRNA/protein-coding RNA pairs. One of the protein-coding RNAs has two concordantly expressed, neighboring lncRNAs. Each pair is separated from the other genes by a white line. The numbers on top indicate hours after the addition of IFNα. The names of genes are shown to the right, with genes named in this study marked by an asterisk. Genes with two asterisks are novel, previously unannotated transcripts. **(B)** The locus of BST2/BISPR. The direction of transcription and name of each gene is shown on top. Exons are shown as solid gray (BST2 and MVB12A) and black (BISPR) rectangles, with introns represented as thin broken lines. The numbers at the bottom of introns and exons indicate size in nucleotides. The distance between the two transcription start sites are shown and the position of important elements are marked. Single-headed arrows on top of exons 2 and 3 of BISPR mark the site of PCR primers used in the majority of the studies described in this work. **(C)** Schematic representation of the density of RNA-seq reads mapping to the BISPR locus. The intron/exon structure of BISPR is shown on top. Numbers at the bottom indicate the position of the locus on chromosome 19. Numbers to the left refer to the time point after the addition of IFNα at which the sample was taken for high-throughput transcriptome analysis. **(D)** Strand-specific RT-PCR confirms the divergent orientation of BISPR and BST2 transcription. F and R refer to forward or reverse primers (relative to the direction of transcription of the analyzed gene). The identity of the primers that were used in reverse transcription reactions are shown below each lane. The large numbers on the Y axis reflect the very low basal expression level of the two RNAs.

To investigate the extent of lncRNA-mediated regulation of nearby protein-coding genes in the IFN response, we took advantage of a high-throughput transcriptome analysis dataset that we had generated using IFN-treated human primary hepatocytes (15). We analyzed the dataset for pairs of protein-coding and non-coding genes that were located within 2 kb of each other and showed robust differential expression in response to IFNα treatment. Using the GENCODE v19 database of putative annotated lncRNAs, we identified nine lncRNA/protein-coding RNA pairs that satisfied these criteria (Figure 1A, Table S1 in Supplementary Material). We also analyzed the data for the presence of novel transcripts that changed expression in response to IFNα and were located in proximity of an IFN-responsive protein-coding gene. We identified two previously unannotated, long transcripts that met our criteria (Figure 1A, RNAs with double asterisks, Table S1 in Supplementary Material). Except two RNAs, which were intronic or positioned in tandem with their protein-coding partner [USP18-IT1 and NRIR/lncRNA-CMPK2 (15), respectively], the other identified transcripts were positioned in a head-to-head, divergent manner compared to their paired protein-coding genes and likely originated from bidirectional promoters (Table S1 in Supplementary Material). Interestingly, one of the identified protein-coding genes was BST2 (Tetherin), which codes for a known IFN-induced protein that blocks the release of enveloped viral particles from infected cells (36–39). In addition to its role as an effector of the IFN-induced antiviral response, it also has roles in negative feedback regulation of IFN production and other immunomodulatory effects (36–40). BST2 was positioned in a head-to-head divergent orientation with an annotated, previously un-studied putative lncRNA, which we named BISPR (see below) (Figure 1B). Similar to the majority of previously described gene pairs originating from putative bidirectional promoters, BST2 and BISPR, together with the rest of gene pairs identified in this study, showed concordant expression in response to IFN stimulation (Figure 1A) (21, 22). The annotated transcription start site (TSS) of BST2 and BISPR were ~250 bp apart, similar to the distance reported for most bidirectionally transcribed gene pairs (22, 30) (Figure 1B).

### Coordinated expression of BST2 and BISPR from a bidirectional promoter

We analyzed the sequence of the putative bidirectional promoter of BST2 and BISPR. Similar to the majority of other bidirectional promoters, it lacks a TATA box, but has a CCAAT promoter element (Figure 1B) (29, 30). Analysis of the ChIP-seq data available in public databases provided evidence for association of CCAAT-binding proteins NF-YA, NF-YB, and CEBPB, and additional transcriptional regulatory factors frequently found to bind to bidirectional promoters such as YY1, c-Myc, NFkB, MAX, and GATA3 (Figure S1 in Supplementary Material) (29, 30, 41). Unlike most reported bidirectional promoters, the BST2-BISPR promoter lacks CpG islands, however, it has been shown that bidirectional promoters use a variety of core promoter elements and are not necessarily dependent on CpG islands for promoter function (42). We also analyzed the sequence of BISPR for decay-inducing and stabilizing elements. It has been shown that the vast majority of transcripts resulting from bidirectional promoter transcription are short, unstable transcripts, which are committed to decay due to the early occurrence of polyadenylation signal motifs (AWTAAA, where W is either A or T) and the absence of stabilizing U1 snRNA-binding sequences (43, 44). While BISPR lacked AWTAAA motifs in its first ~1000 nt, it had multiple copies of U1-binding sequences and splicing events occurring in its first 500 bases, which explained its stability. Most interestingly, the shared promoter region of BST2 and BISPR falls within overlapping H3K4me1, H3K4me3, and H3K27ac histone modification marks, which are characteristic of transcribed, active enhancers (Figure S1 in Supplementary Material) (24, 45).

**Figure 2:**
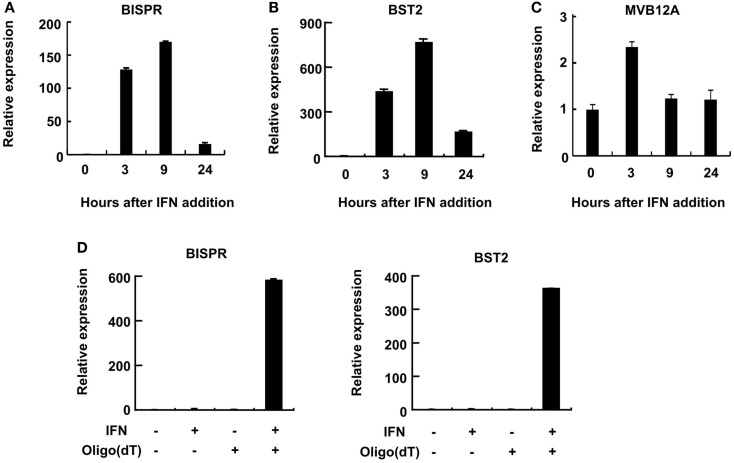
**BST2 IFN-stimulated positive regulator is a polyadenylated RNA and shows concordant expression with BST2**. **(A–C)** RT-qPCR assays show the extent of induction of BISPR, BST2, and MVB12A in response to IFNα stimulation. The identity of the RNA analyzed in each panel is shown on top. The large numbers at the Y axis of A and B reflect the very low basal expression level of the RNAs. **(D)** BISPR and BST2 are polyadenylated RNAs. Plus and minus signs indicate the presence or absence of the shown ingredients in the cell culture medium or reverse transcription reactions prior to qPCR assays.

Consistent with the induction of expression of BST2 and BISPR in response to IFN stimulation, the putative bidirectional promoter contains an ISRE element and ChIP data in public databases showed the binding of STAT1 and STAT2 proteins to this region (Figure 1B and Figure S1 in Supplementary Material). Analysis of the RNA-seq data in publicly available databases provided evidence for concordant transcription in BST2 and BISPR loci (Figure S1 in Supplementary Material), similar to what we had observed in our RNA-seq analysis. Pattern of RNA-seq reads at the locus of BISPR indicated the presence of an efficiently spliced, multi-exonic transcript with four exons and three introns (Figures 1B,C and Figure S1 in Supplementary Material). To confirm the presence of this transcript, we performed strand-specific RT-PCR assays using primers that flanked an exon–exon junction and thus, could only detect spliced, fully mature transcripts (Figure 1B, single-headed arrows). We observed evidence for the presence of an IFN-induced transcript in the expected orientation, while no RNA could be detected originating from the opposite strand (Figure 1D). As a control, we performed an identical analysis on BST2, which showed the presence of an IFN-induced transcript in the annotated locus of BST2 in opposite orientation to BISPR (Figure 1D). We further confirmed the IFN-induced expression pattern of BISPR and BST2 in Huh7.5 hepatocytes, which showed an overall similar pattern of IFN-mediated time course of transcriptional induction between BST2 and BISPR (Figures 2A,B). In contrast, the expression of MVB12A gene, which is located in proximity of BISPR (Figure 1B) did not show a significant induction (Figure 2C).

### BISPR is a spliced and polyadenylated long non-coding RNA

**Figure 3:**
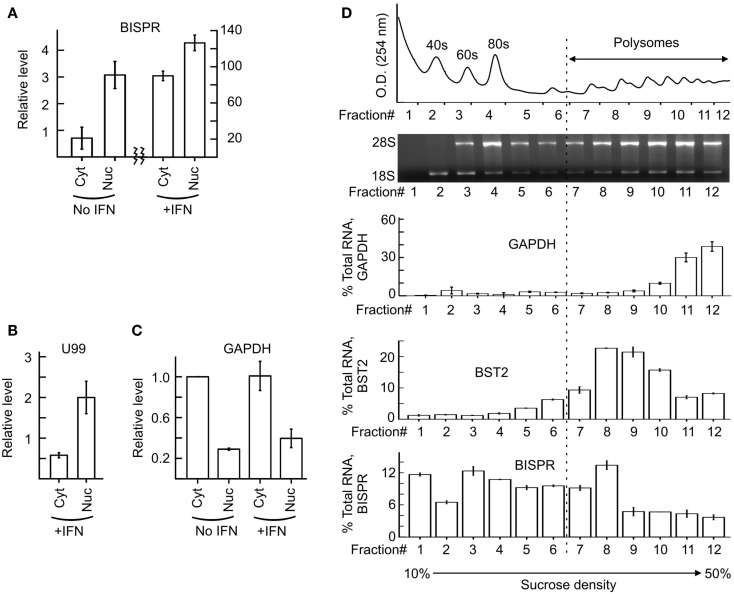
**Subcellular localization of BISPR before and after IFN stimulation**. **(A)** RT-qPCR assays for detection of BISPR performed on RNA extracted from nuclear and cytoplasmic fractions obtained before and after IFNα addition. Note the two Y axes. **(B,C)** Detection of level of U99 snoRNA [**(B)**, nuclear marker] and GAPDH [**(C)**, cytoplasmic marker] by RT-qPCR in subcellular fractions analyzed in panel **(A)**. The presence of a significant level of U99 in cytoplasmic fractions indicates the leakage of nuclear RNAs into the cytoplasmic fraction preparation. **(D)** Polysome profile analysis of BISPR. The top two panels show the position of the ribosomal RNA and RNP complexes. The bottom three panels are RT-qPCR assays to determine the level of GAPDH (top), BST2 (middle), and BISPR (bottom) in each fraction, expressed as percentage of total signal detected in all fractions combined. The number of fractions are shown at the bottom.

We could show that BISPR, similar to BST2, is a polyadenylated message (Figure 2D) and confirmed the presence and location of the splice junctions by sequencing its cDNA (data not shown). Next, we determined the ends of the mature BISPR transcript using 5′ and 3′ RACE assays (Figures S2, S3 in Supplementary Material), which showed minor differences at TSS and 3′ cleavage sites compared to the annotations in public databases. Phylogenetic analysis indicated that the region between the TSS of BST2 and BISPR and the first two exons and parts of the last exon of BISPR were conserved among mammals, with the third exon of BISPR only showing conservation among primates (Figure S4 in Supplementary Material). We analyzed the subcellular localization of BISPR, which showed that in the absence of IFN it was predominantly nuclear, while after IFN induction the RNA was present in both compartments (Figures 3A–C). To determine if BISPR had any protein-coding capacity, we analyzed the sequence of the RNA for the presence of ORFs, which showed the presence of five potential ORFs that were between 36 and 49 amino acids in length (Figure S5 in Supplementary Material). The position of ORFs within BISPR was not conducive to efficient translation, as they either ended before the last exon–exon junction or had upstream ORFs, which will result in activation of NMD-mediated decay pathways if the RNA is subjected to translation (Figure S5 in Supplementary Material). We further analyzed the sequence of the ORFs for potential protein-coding capacity. Analysis of their conservation level indicated that neither of the ORFs showed enhanced conservation compared to the rest of the exonic or even intronic sequences in BISPR (Figures S6A–D in Supplementary Material). The ORFs lacked the Kozak consensus sequence, and showed frequent presence of frameshift-inducing small insertions and deletions at a frequency that was not different from the adjacent, non-ORF sequences (Figures S6A–D in Supplementary Material). Further, the sequence variations between species resulted in a large ratio of non-synonymous to synonymous changes in ORFs, which indicated lack of conservation of the sequence of the resulting peptides even among closely related species (Figures S6A–D in Supplementary Material). To complement and experimentally confirm the computational analysis of the protein-coding capacity of BISPR, we asked whether the cytoplasmic fraction of BISPR associated with polysomes. As can be seen in Figure 3D, unlike GAPDH and BST2, which showed clear enrichment in polysome fractions, the cytoplasmic BISPR RNA showed a more or less uniform distribution across the sucrose gradient, with reduced levels in heavier fractions. This pattern indicates that the distribution of BISPR RNA is independent of the location of ribosomal complexes in the gradient and is likely governed by the set of proteins and other cellular factors that associate with this RNA. Taken together, these results indicated that BISRP was not likely to code for functional peptides and thus, was a spliced and polyadenylated, long non-protein-coding RNA.

### BISPR is induced through IFN-mediated activation of JAK–STAT pathway in diverse cell types

**Figure 4:**
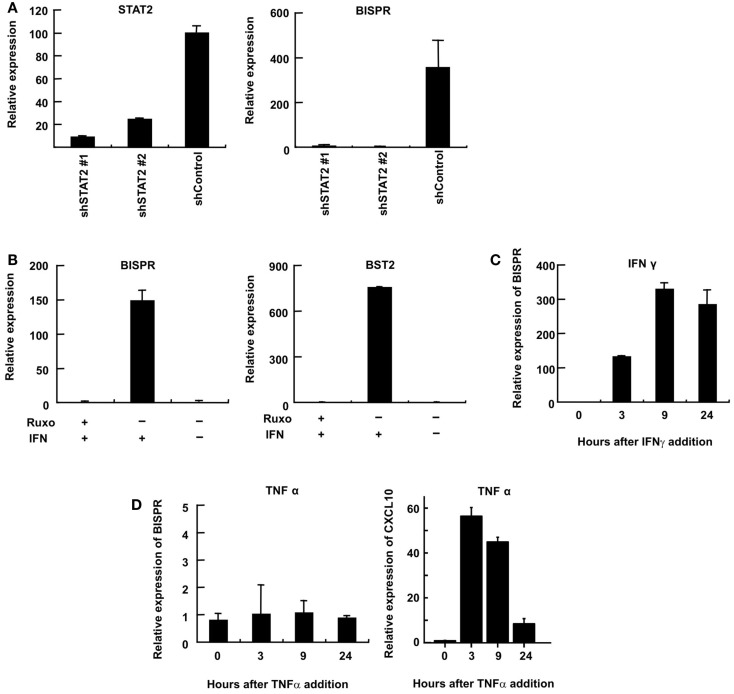
**Interferon-mediated induction of expression of BISPR occurs through the JAK–STAT pathway**. **(A)** RT-qPCR assays to measure the level of STAT2 (left) and BISPR (right) to show the level of knock down of STAT2 and the level of induction of BISPR after IFN stimulation in STAT2 knock down cells, respectively. The identity of the shRNA construct used in each sample is shown below the graph. **(B)** Assays to determine the level of IFN-mediated induction of BISPR (left) and BST2 (right) before and after treatment with the JAK-inhibitor, ruxolitinib (Ruxo). Plus and minus signs indicate the added or omitted ingredients. **(C)** BISPR RNA is induced in response to IFNγ stimulation. **(D)** BISPR is not induced in response to TNFα stimulation (left), while a known TNFα-responding ISG, CXCL10, is strongly induced as a result of TNFα treatment (right).

**Figure 5:**
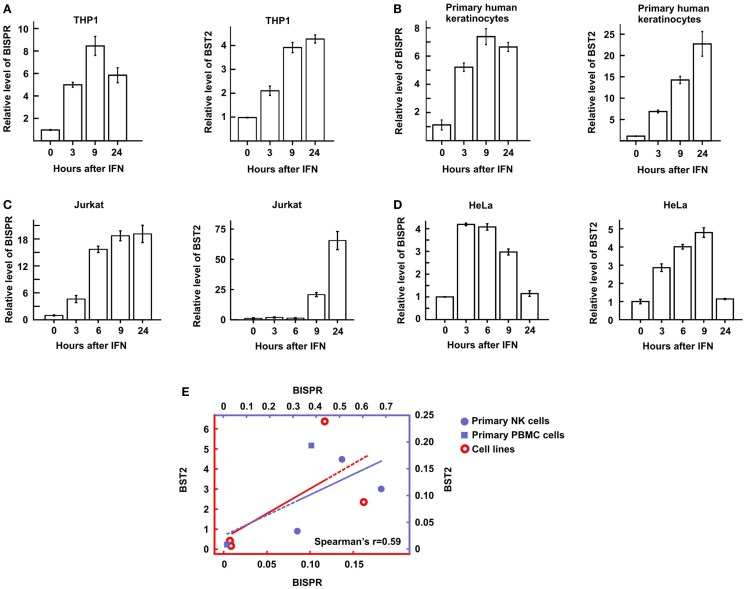
**BST2 IFN-stimulated positive regulator is induced in diverse cell types in response to IFN and shows a concordant expression pattern with BST2**. **(A–D)** Time course of IFNα-mediated induction of BISPR (left) and BST2 (right) in THP1 cells [monocyte origin, **(A)**], primary human keratinocytes **(B)**, Jurkat T cells **(C)**, and HeLa cells [cervical origin, **(D)**] measured by RT-qPCR. The identity of cells being analyzed is shown on top. **(E)** Correlation of the level of BISPR and BST2 in the four cells lines shown in **(A–D)** (empty circles), and NK and PBMC cells obtained from human donors (solid circles and squares, respectively). Note the two sets of axes and trendlines, which mark the values of samples of the corresponding color. Vertical and horizontal axes correspond to BST2 and BISPR values, respectively. The Spearman correlation coefficient for the entire sample group is shown at the bottom right of the graph.

We could show that BISPR was a bona fide ISG as blocking the JAK–STAT pathway by shRNAs against STAT2 or the use of JAK-inhibitor ruxolitinib prevented its IFN-mediated up-regulation (Figures 4A,B). BISPR was induced in response to both type I and type II IFNs (IFNα and IFNγ, respectively), however, despite the presence of NFkB binding sites on the shared BISPR–BST2 promoter, it did not show a transcriptional up-regulation after treatment with TNFα (Figures 4C,D). Since BST2 is known to mainly show antiviral activity against several viral families that include retroviruses but neither of the major hepatotropic viruses (36), we asked if IFN-mediated induction of BISPR could also occur in cells involved in retroviral infections such as immune cells. We could show that IFN stimulation of human THP1 monocytic cells and Jurkat T cells resulted in induction of BISPR (Figures 5A,C). In addition, we tested other cell types including primary human keratinocytes and HeLa cells (cervical epithelium origin) and observed that BISPR was induced in response to IFNα in these cells (Figures 5B,D). These results suggested that the IFN-mediated induction of BISPR expression was not restricted to a certain cell type, but that the RNA was a general IFN-induced factor. Importantly, analysis of IFN-induced BST2 expression in parallel showed that the pattern of induction of BISPR showed concordance with BST2 in all cell types tested (Figures 5A–D, see also Figures 2A,B), confirming the coordinate regulation of the two RNAs. To further analyze the correlation of expression of the two RNAs in immune system-derived cells, we measured the level of BISPR and BST2 in primary human NK and PBMC derived from healthy human donors (Figure 5E). While the small number of samples (*n* = 3 for NK cells and *n* = 2 for PBMC) prevents us from deriving statistically significant conclusions from these data, the level of BST2 and BISPR in both primary cells and the cell lines studied above showed a modest correlation, with a Spearman’s correlation coefficient of 0.59 for both groups (Figure 5E). Since THP1 cells represented a more physiologically relevant study model than Huh7.5 hepatocytes for the study of function of BISPR, all subsequent studies were performed in this cell line.

### lncRNA BISPR is a positive regulator of the expression of BST2

**Figure 6:**
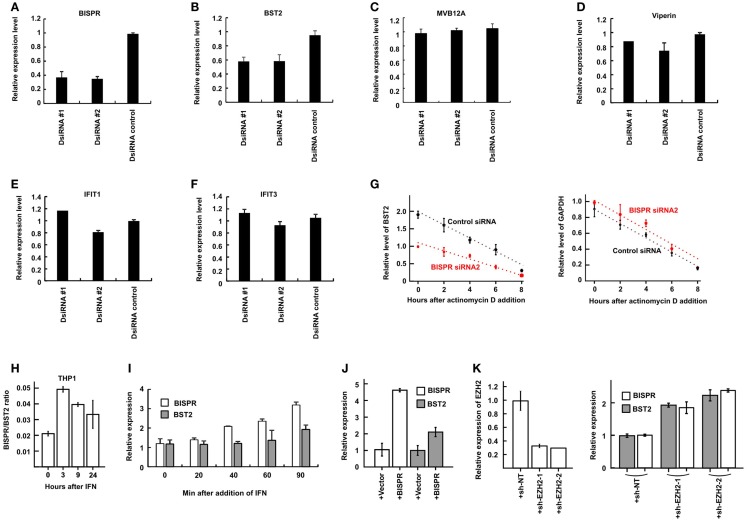
**BISPR is a positive regulator of BST2 expression**. **(A–F)** RT-qPCR assays to determine the relative cellular level of transcripts shown on top of each panel in THP1 cells treated with BISPR-targeting siRNAs (DsiRNA #1 and #2) and control siRNA. Statistical analysis of the control siRNA and BISPR-targeting siRNA groups yielded *p*-values <0.05 for panels A and B. The differences between these groups in panels C through F were not significant. **(G)** The relative level of BST2 (left) and GAPDH (right, analyzed as control) are measured at several time points after the addition of actinomycin D to block transcription in cells transfected with a control siRNA (data shown in black) or BISPR-targeting siRNA (data shown in red). The level of BST2 or GAPDH is measured at each time point using RT-qPCR assays. **(H)** BISPR shows an earlier induction curve in response to IFNα stimulation compared to BST2. The Y axis indicates the ratio of the level of BISPR to BST2 transcripts (delta Ct) at each time point after IFN stimulation. **(I)** Early time points after IFNα stimulation of THP1 cells confirm that the induction of expression of BISPR precedes that of BST2. Cellular RNA has been extracted at the indicated time points and analyzed in parallel to determine the level of BST2 and BISPR using RT-qPCR assays. **(J)** RT-qPCR measurement of the level of BISPR and BST2 in THP1 cells stably overexpressing BISPR from a transgene. + Vector and + BISPR indicate cells stably transfected with a control, empty vector or the BISPR construct, respectively. **(K)** The efficiency of shRNA-mediated knock-down of EZH2 (left panel) and the level of BISPR and BST2 in control and knock down THP1 cells (right panel) are shown, as determined by RT-qPCR. + sh-NT and + sh-EZH2-1 and 2 indicate cells stably transfected with a non-targeting shRNA or one of the two EZH2-targeting shRNA constructs, respectively.

As mentioned above, study of a number of lncRNA/protein-coding RNA pairs resulting from bidirectional promoters has shown a role for the lncRNAs in regulation of expression of their protein-coding “twins.” To directly investigate this possibility in BST2/BISPR pair, we knocked down the expression of BISPR using siRNAs. In order to prevent the possibility of siRNA-mediated interference with the function of the enhancer or promoter elements, we ensured that the siRNAs were designed against sequences in the last two exons of BISPR and far from the promoter and the enhancer regions (see Materials and Methods). Interestingly, siRNAs which reduced the level of BISPR also reduced the level of BST2 (Figures 6A,B), suggesting that transcription of the BISPR RNA is required for efficient transcription of BST2 mRNA. To ensure that the observed down-regulation did not result from a general transcriptional silencing of the locus, we measured the expression level of MVB12A, which is located a short distance downstream of BISPR and close to the region targeted by the siRNAs. However, the transcription of MVB12A showed no change in siRNA-treated cells (Figure 6C, see also the BISPR overexpression study below). To determine if any other ISGs beyond BST2 were affected by BISPR knock down, we tested the expression level of several other ISGs in siRNA-treated cells, which did not show significant changes in response to siRNAs (Figures 6D–F). Thus, knock down of BISPR seemed to result in specific down-regulation of BST2.

We asked whether the observed reduction in the steady-state level of BST2 mRNA resulted from modulation of transcription of this RNA or a change in its stability. To this end, we blocked transcription in siRNA-treated cells using actinomycin D treatment and measured the level of BST2 mRNA at several time points (Figure 6G). We observed a slower, rather than faster, decay rate for BST2 mRNA in siRNA-treated cells, which indicated that the reduced stability of BST2 mRNA was not responsible for the lower level of BST2 mRNA in these cells. Thus, knock down of BISPR resulted in transcriptional down-regulation of BST2. The above results suggested that expression of BISPR was required for the efficient transcription of BST2 locus and thus, this RNA seemed to function as BST2 IFN-Stimulated Positive Regulatory RNA (BISPR).

Interestingly, comparison of the time course of induction of BISPR and BST2 in response to IFN indicated a faster rate of induction of BISPR compared to BST2 in all tested cell types (Figure 6H, compare Figures 5A–D). To further substantiate this observation, we took early time points after IFN stimulation in THP1 cells and monitored the level of BST2 and BISPR RNA in parallel. Intriguingly, while BST2 did not show a significant induction until 90 min after IFN stimulation, the level of BISPR showed a significant rise after only 40 min (Figure 6I), suggesting that transcriptional activation of the locus is initiated by induction of the expression of BISPR RNA. To further confirm this possibility, we forcefully overexpressed the fully spliced BISPR RNA from a stably transfected construct under the control of the constitutive CMV promoter (Figure 6J). Interestingly, overexpression of BISPR from the transgene resulted in up-regulation of expression of the endogenous BST2 gene (Figure 6J), indicating that BISPR RNA itself, rather than the impact of its transcription from a locus adjacent to BST2, is responsible for regulation of expression of BST2. As mentioned above, many lncRNAs are thought to act by regulation of chromatin state through binding and recruitment of various repressor or activator chromatin modifying factors (e.g., PRC2 and WDR5, respectively) to their regulatory loci (7, 46) and it is possible to envision that upon IFN stimulation, BISPR may act by recruiting an activator protein to its shared locus with BST2 to counteract a constitutive repressive chromatin state. As a first step toward investigating this possibility, we asked whether the transcriptional activity of BISPR/BST2 locus under basal, unstimulated conditions is regulated by one of the known repressive chromatin modifying complexes. Using shRNAs targeting the methyltransferase component of PRC2, EZH2, we could show that even a modest reduction in the level of EZH2 can result in up-regulation of both BISPR and BST2 (Figure 6K). While defining the mechanism of function of BISPR and identification of its interacting factors require extensive work beyond the scope of the current manuscript, these preliminary results suggest that counteracting the repressive action of PRC2 at this locus can be a potential mechanism through which BISPR can perform its regulatory function.

## Discussion

We have shown that the expression of several IFN-induced protein-coding genes show a strong correlation with a lncRNA gene located in their vicinity. Analysis of one such lncRNA/protein-coding RNA pair suggested that they originate from a bidirectional promoter, thus explaining their coordinated regulation. Interestingly, it has recently been shown that the presence of protein-coding/lncRNA pairs resulting from bidirectional promoters is frequently seen among transcriptionally regulated protein-coding transcripts (25). This, in turn, raises the possibility that the presence of a promoter-sharing lncRNA provides additional means for fine-turning the regulation of expression of such protein-coding RNAs. Indeed, in some studied cases, the expression of protein coding genes is regulated by their promoter-sharing lncRNA, with RNAi-mediated knock down of the lncRNA resulting in up-regulation of expression of the protein-coding gene (31, 32). Our analysis of the BST2/BISPR pair provides a new example for such regulation, albeit in the opposite direction, as knock down and overexpression of BISPR led to transcriptional down- and up-regulation of BST2, respectively, suggesting an activating role for BISPR in regulation of BST2 expression.

While several studied lncRNAs have an activating impact on their neighboring protein-coding genes (47, 48), divergent transcription from a bidirectional promoter provides unique opportunities for regulation. Divergent transcription may induce local chromatin remodeling, which may be a pre-requisite for the activation of the downstream gene, as has been shown for adjacent genes (49). Also, divergent transcription can induce negative upstream supercoiling of DNA and thus, promote upstream transcriptional initiation (23). Further, the nascent or fully transcribed RNA may recruit chromatin remodeling or transcriptional complexes to its site of transcription and thereby influence the transcriptional activity of the locus (50, 51). We have shown that in the case of BISPR/BST2 pair, expression of BISPR is the first to be induced by activating stimuli, and importantly, that forced overexpression of BISPR from a transgene (which is unlikely to be integrated close to the locus of the endogenous BISPR/BST2 pair in all overexpression clones tested) results in up-regulation of BST2. These results strongly point to the third scenario described above, in which the mature transcribed lncRNA mediates the recruitment of chromatin modifying factors or activating transcriptional complexes to its site of transcription, however, the other two, cis-acting mechanisms described above may also contribute to the regulatory function of BISPR.

As mentioned before, analysis of chromatin modification patterns suggests that the BISPR/BST2 locus overlaps an enhancer region. It is known that many, if not all, enhancers are bidirectionally transcribed into functionally required enhancer-associated RNAs (eRNAs) (52–54). It has been proposed that either the eRNA transcripts themselves or the act of transcription contributes to the function of enhancers (55). While the efficient formation of the enhancer–promoter contact is thought to require the interaction of eRNAs with the mediator complex and cohesin (45, 55), eRNAs may help establish chromatin accessibility, thus enhancing transcription (56). Based on the existing literature, however, the majority of eRNAs are much shorter than BISPR and BST2, and are not spliced or polyadenylated (52–54). In cases where eRNAs were polyadenylated, they were transcribed uni-directionally rather than bidirectionally. While BISPR is certainly not a classical eRNA, it is perceivable that it may also participate in regulation of expression of hitherto un-identified distant target genes as an eRNA. There is also the remote, but nonetheless existing, possibility that the BISPR/BST2 pair may have evolved from an ancestral bidirectional enhancer-associated promoter and transcription of either of the RNAs may affect the ability of the enhancer to regulate any distant targets that it may have. Interestingly, an example of bidirectional lncRNA/protein-coding RNA transcription originating in an enhancer has recently been described, in which the transcription of the lncRNA and the ability of the enhancer to induce transcription in a downstream gene seemed to be coordinated (57). Taken together, these findings provide a novel example of stable bidirectional transcription from transcribed enhancer loci and point to the presence of RNA-mediated regulatory networks involving bidirectionally transcribed lncRNA/protein-coding RNA pairs. Considering the abundance of lncRNA/protein-coding RNA pairs originating from bidirectional promoters in the human genome, it is possible that the lncRNA-mediated transcriptional regulation described in this work contributes to the regulation of expression of additional protein-coding genes in the IFN response and other cellular processes.

## Author Contributions

Saba Valadkhan and Hiroto Kambara designed the study. Saba Valadkhan performed the computational analysis of the data. Hiroto Kambara and Lalith Gunawardane performed the experiments, except as noted below. Elizabeth Zebrowski, Lenche Kostadinova and Donald D. Anthony collected blood samples from human donors and prepared NK and PBMC fractions for analysis. Raul Jobava, Dawid Krokowski and Maria Hatzoglou optimized the sucrose gradient fractionation of polysomal complexes, performed gene expression assays and analyzed the data and prepared the relevant figure. Saba Valadkhan re-analyzed all data for accuracy and wrote the manuscript. All authors read the manuscript and commented on it prior to final submission.

## Conflict of Interest Statement

The authors declare that the research was conducted in the absence of any commercial or financial relationships that could be construed as a potential conflict of interest.

## Supplementary Material

The Supplementary Material for this article can be found online at http://www.frontiersin.org/Journal/10.3389/fimmu.2014.00676/abstract
