# Supplementary material for: Regulation of Interferon-Stimulated Gene BST2 by a lncRNA Transcribed from a Shared Bidirectional Promoter
Source: Front Immunol. 2015 Jan 30;5:676. doi: 10.3389/fimmu.2014.00676 (PMC4311693; doi:10.3389/fimmu.2014.00676)
Supplement: Supplementary file 2 [file Image_1.PDF]

Supplementary material for

Regulation of Interferon-stimulated gene BST2 by a lncRNA transcribed from a shared bidirectional promoter

Hiroto Kambara[1,*], Lalith Gunawardane[1,*], Elizabeth Zebrowski[2], Lenche Kostadinova[2], Raul Jobava[3], Dawid Krokowski[3], Maria Hatzoglou[3], Donald D. Anthony[2] and Saba Valadkhan[1,**]

[1]Department of Biochemistry, Case Western Reserve University School of Medicine, Cleveland, OH, USA

[2]Divisions of Infectious and Rheumatic Diseases, Department of Medicine, School of Medicine, Case Western Reserve University, Cleveland, OH 44106, USA

[3]Department of Nutrition, Case Western Reserve University School of Medicine, Cleveland, Ohio 44106

* These authors have contributed equally to this work

Correspondence:

Dr. Saba Valadkhan

Case Western Reserve University School of Medicine

Department of Molecular Biology and Microbiology

10900 Euclid Avenue, Wood W210a,

Cleveland, OH 44106, USA

Saba.valadkhan@case.edu

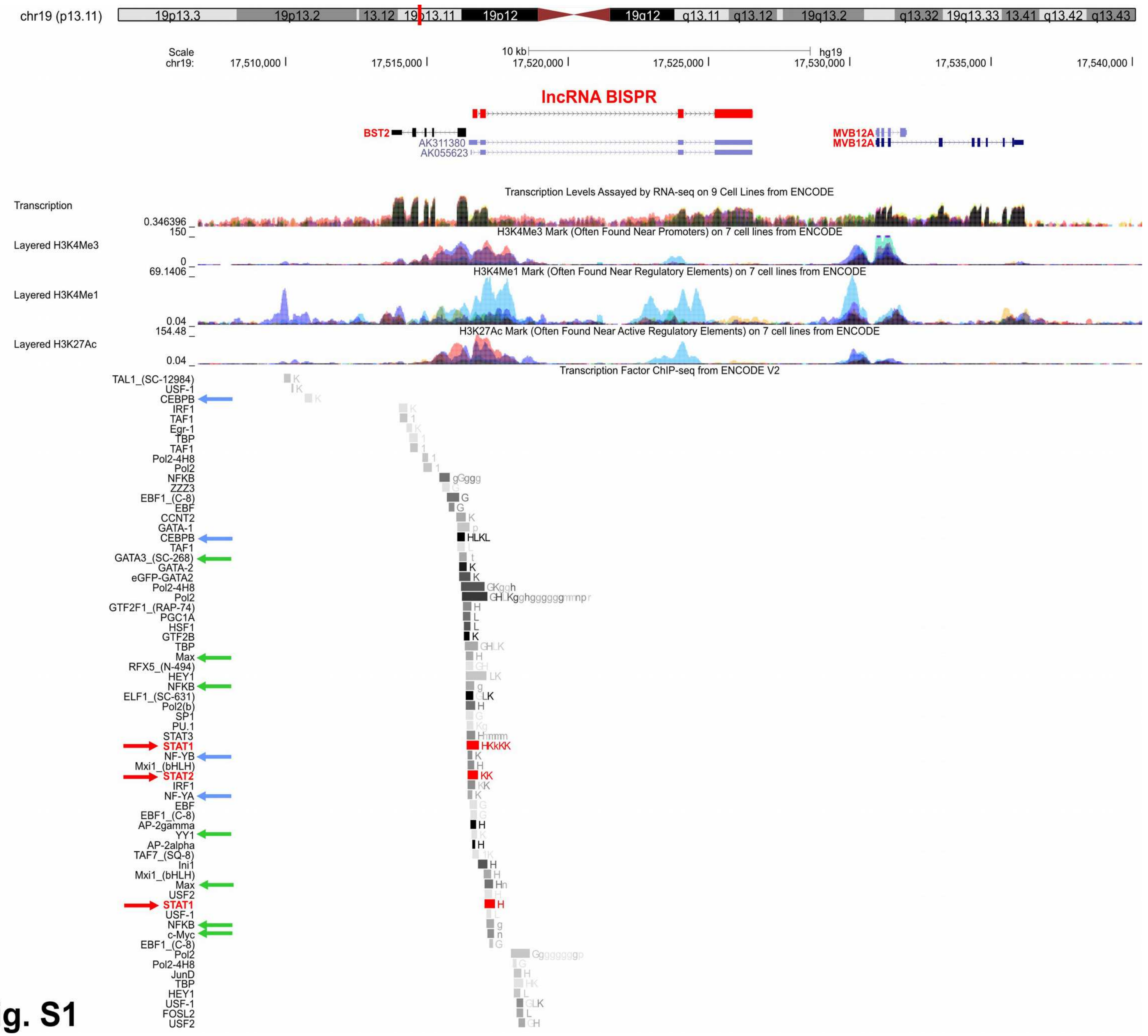
chr19 (p13.11)
19p13.3
19p13.2
13.12
19p13.11
19p12
19q12
q13.11
q13.12
19q13.2
q13.32
19q13.33
13.41
q13.42
q13.43
Scale
chr19:
10 kb
hg19
17,510,000
17,515,000
17,520,000
17,525,000
17,530,000
17,535,000
17,540,000
lncRNA BISPR
BST2
AK311380
AK055623
MVB12A
MVB12A
Transcription Levels Assayed by RNA-seq on 9 Cell Lines from ENCODE
Transcription
0.346396
150
H3K4Me3 Mark (Often Found Near Promoters) on 7 cell lines from ENCODE
Layered H3K4Me3
0
69.1406
H3K4Me1 Mark (Often Found Near Regulatory Elements) on 7 cell lines from ENCODE
Layered H3K4Me1
0.04
154.48
H3K27Ac Mark (Often Found Near Active Regulatory Elements) on 7 cell lines from ENCODE
Layered H3K27Ac
0.04
Transcription Factor ChIP-seq from ENCODE V2
TAL1_(SC-12984)
USF-1
CEBPB
IRF1
TAF1
Egr-1
TBP
TAF1
Pol2-4H8
Pol2
NFKB
ZZZ3
EBF1_(C-8)
EBF
CCNT2
GATA-1
CEBPB
TAF1
GATA3_(SC-268)
GATA-2
eGFP-GATA2
Pol2-4H8
Pol2
GTF2F1_(RAP-74)
PGC1A
HSF1
GTF2B
TBP
Max
RFX5_(N-494)
HEY1
NFKB
ELF1_(SC-631)
Pol2(b)
SP1
PU.1
STAT3
STAT1
NF-YB
Mxi1_(bHLH)
STAT2
IRF1
NF-YA
EBF
EBF1_(C-8)
AP-2gamma
YY1
AP-2alpha
TAF7_(SQ-8)
Ini1
Mxi1_(bHLH)
Max
USF2
STAT1
USF-1
NFKB
c-Myc
EBF1_(C-8)
Pol2
Pol2-4H8
JunD
TBP
HEY1
USF-1
FOSL2
USF2

Fig. S1

Fig. S1. The locus of BST2/BISPR. The chromosome is schematically shown on top. The position of the locus on chromosome 19 and the genomic structure of BISPR, BST2 and MVB12A are shown. The identity of each track or bound protein is shown to the left. Red arrows mark the transcription factors that mediate the IFN-stimulated transcriptional upregulation. Green arrows point to factors frequently found to bind bidirectional promoters. Blue arrows mark factors that bind the CCAAT promoter element. The data is taken from the UCSC genome browser.

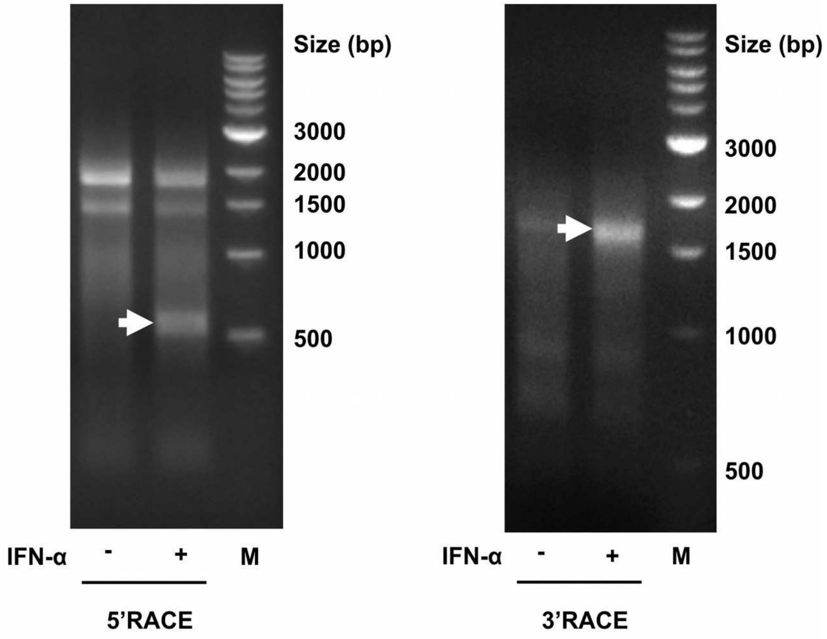

Fig. S2

Fig. S2. 5' and 3' RACE assays show the presence of a single IFN-induced band. The band was purified and sequenced to determine the 5' and 3' ends of BISPR.

LncRNA BISPR (human)
1942 nts long spliced RNA
Chr19 (p13.11):17,516,632-17,526,539

```
GGTGCCAGCC AACTGTGGCT GCCTGGGACA CTCCATCTTA AGTCCCTGTG CCTCTGCTGT     60
GTGTTACTGA GTGCCTAGGC CGTGCCAGCC TGTATTCATC TGTACTATGA CCTGAAGAGG    120
CAGAGGCCAT CACTGTTGGT CCGGTCTCCA CCTGGGGAAA CTGAGGTTGC ACA/GTGTCTC   180
TGTGGTGACG AGCAGGGCTT CATCCAGTGC CTCTGTCCCC ACCGAGGGGA CTATGGGAGA    240
CATGGAGGGT GTGTGAGCAA CAGGTGAGAC TGGAGCCAGC TGAAAACTGG GAGACCGACC    300
CAGCCAACAA ACAATGTCGG TCTCTGTCTT GGCACCTGCA GGAAACAAGC TCCTACTTCC    360
AGAAAAAGTG CTCCTGGGAC TCCAGGATAC CAG/GCATCTG GGTAAGCTAC AATGCTTAAC   420
CACTTAACAC AATCAGGAAG CAACAGCCAT GCATTCGGGA AAGGAACTTC AGTGTTGTGT    480
GGCTCAGTCT CCAGACCTAA CTTTCCCTTT GGTACAGACG CGGTGGTACA TGCCTGTAAT    540
CCCAACACTT AGGCAGGAGG ATCACTCGAG CCCAGAAGAT CAAGACTAGT GTGAGCAACA    600
AA/ATCCTTCC CTGGCACCGG TTCCAGGAGG ACCTCGTTCT TAGGCAGCAT CTGTGTCTCT   660
GTTTTGTAAG ACACACAGAG TATCCTTAAC CCACTGCAGA TATTTCCCTG GGTCCATGGT    720
CAATCAACAC CACCTTTCAG TCTCCAGCCT CTTAACCAGC CCAGGGCTGA GTGTTGTTTA    780
TAGTATTAAT ATGTTTCTGG CTGGGCTCAG TGGCTCATGC CTgTAATCCC GGCACTATGG    840
GAGGCTGAGG CAGGCGGATC ACTTGAGGTC AGGAGTTCAA GACCAGCCTG GCCAACATaG    900
TAAAAcCCCG TCTCTACTAA AAATACAAAA ATGAGCCGGG TGTGGTGGCG TGTGTCTATA    960
ATCCCAGCTA CTCGGGAGGC TGAGGCAGgA GAATTGCTTG AACCTGGGAG GTGGAGGCTA   1020
CAGTGAGCTG AGATCATGCC ACTGCACTCC AGCCTGGGCA AGAAAGTGAG ACTCCATCTC   1080
AAAACAAAGA AACAAACAAA GTATTAATAT GTTCCCCTTG ATCAGTGGGG TGGTTCACCC   1140
TTGTAATCCC AGCACTTTGG GAGGCAGAGG CTGGCGGATC GCTTGAGTCC AGGAGTTCGA   1200
GACCAGCCTG GGTGAACTAG CAAAACCCTG CCTGTACTAA AAATACAAAA ATTAACTGGG   1260
CTTGGTGGTG CGAGCCTGTA ATCCCAGTGG AGGCTGAGAC AGGAGAATCC TTTTGTAATG   1320
CCTgACCTTG TTTttttact aaccctattt taaccttgTT TTTACTAACC CTATTTTTAA   1380
ATTTTCCCTT TTTGTCTCCT TAATTACCTA GCCTTGTTTC CCATATGAGT AGACTCTCCC   1440
TTAGCTGGGA AAGCCTGACG AACGCCATCT GGCCCCTTGA TTTACAAGAC ATTAAGGGCT   1500
ACTTACCCAA CCCCCTTCCT CAAAgAGTTA ACCTGCGTAA GCAGATCCTC AGCATTTCAA   1560
AGGAGCCCAA TTAGCTGATA AGGTACTGGA ACAAACAATG TACGAAGTTC CCAGGATTTT   1620
GCTCAAAAGC ATAACAACAT AAAGCCTTGA GTCTGTGTCT GGCATAGCAT CCATATCTAA   1680
CTCTTATGAA GGATTTAGAG CCCGGCACCT GGTTCCGTTG CTTTTTTTGT AACCATTTGT   1740
CTTTTAAATT GTTTATTTCT CTGTAACCAT TTGTTTTTTG ATTCTTGCAT GTTTTTACTT   1800
CTGTAGAATT ATTGCATTTG AGCTCCGCTC CCCTTCCGAA CCAAGGTATA AAAGTAAATC   1860
AAGCCCCTTC CTCGGGGCCG AGAGAATTTT GGAAAGTCAA GCCTTCTCTT GGCTGCCGGC   1920
TTAAATAAAG GACTCTTAAc TC|GTCTCGAA GTGTGGCGTT TTCTCTAACT CGCTTGGGTA   1942
```

Fig. S3

Fig. S3. The deduced sequence of mature BISPR RNA. The sequence is determined based on the 5' and 3' RACE analyses, and the location of splice sites (shown as a red forward slash) was confirmed by sequencing. The position of the polyadenylation signal is shown in bold, and the cleavage site is marked by a vertical line highlighted in red. The downstream GU-rich element is highlighted in gray.

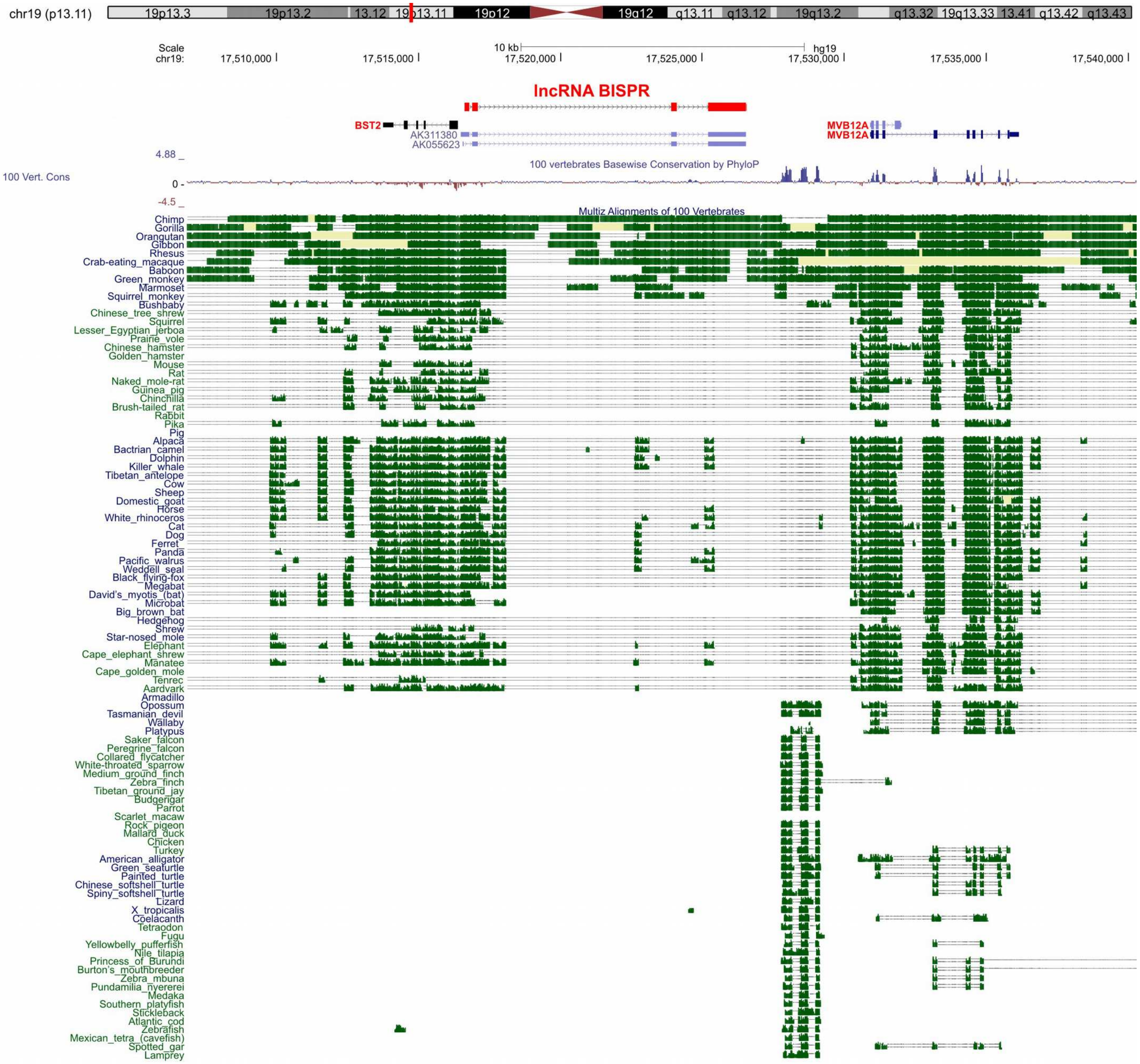
chr19 (p13.11)
19p13.3
19p13.2
13.12
19p13.11
19p12
19q12
q13.11
q13.12
19q13.2
q13.32
19q13.33
13.41
q13.42
q13.43
Scale
chr19:
10 kb
hg19
17,510,000
17,515,000
17,520,000
17,525,000
17,530,000
17,535,000
17,540,000
lncRNA BISPR
BST2
AK311380
AK055623
MVB12A
MVB12A
4.88 _
100 Vert. Cons
0 -
-4.5 _
100 vertebrates Basewise Conservation by PhyloP
Multiz Alignments of 100 Vertebrates
Chimp
Gorilla
Orangutan
Gibbon
Rhesus
Crab-eating_macaque
Baboon
Green_monkey
Marmoset
Squirrel_monkey
Bushbaby
Chinese_tree_shrew
Squirrel
Lesser_Egyptian_jerboa
Prairie_vole
Chinese_hamster
Golden_hamster
Mouse
Rat
Naked_mole-rat
Guinea_pig
Chinchilla
Brush-tailed_rat
Rabbit
Pika
Pig
Alpaca
Bactrian_camel
Dolphin
Killer_whale
Tibetan_antelope
Cow
Sheep
Domestic_goat
Horse
White_rhinoceros
Cat
Dog
Ferret
Panda
Pacific_walrus
Weddell_seal
Black_flying-fox
Megabat
David's_myotis_(bat)
Microbat
Big_brown_bat
Hedgehog
Shrew
Star-nosed_mole
Elephant
Cape_elephant_shrew
Manatee
Cape_golden_mole
Tenrec
Aardvark
Armadillo
Opossum
Tasmanian_devil
Wallaby
Platypus
Saker_falcon
Peregrine_falcon
Collared_flycatcher
White-throated_sparrow
Medium_ground_finch
Zebra_finch
Tibetan_ground_jay
Budgerigar
Parrot
Scarlet_macaw
Rock_pigeon
Mallard_duck
Chicken
Turkey
American_alligator
Green_seaturtle
Painted_turtle
Chinese_softshell_turtle
Spiny_softshell_turtle
Lizard
X_tropicalis
Coelacanth
Tetraodon
Fugu
Yellowbelly_pufferfish
Nile_tilapia
Princess_of_Burundi
Burton's_mouthbreeder
Zebra_mbuna
Pundamilia_nyererei
Medaka
Southern_platyfish
Stickleback
Atlantic_cod
Zebrafish
Mexican_tetra_(cavefish)
Spotted_gar
Lamprey

Fig. S4

Fig. S4. Conservation of the genes at and near the BISPR locus. The chromosome is schematically shown on top. The position of the locus on chromosome 19 and the genomic structure of BISPR, BST2 and MVB12A are shown. The identity of each track or species is shown to the left. The data is taken from the UCSC genome browser.

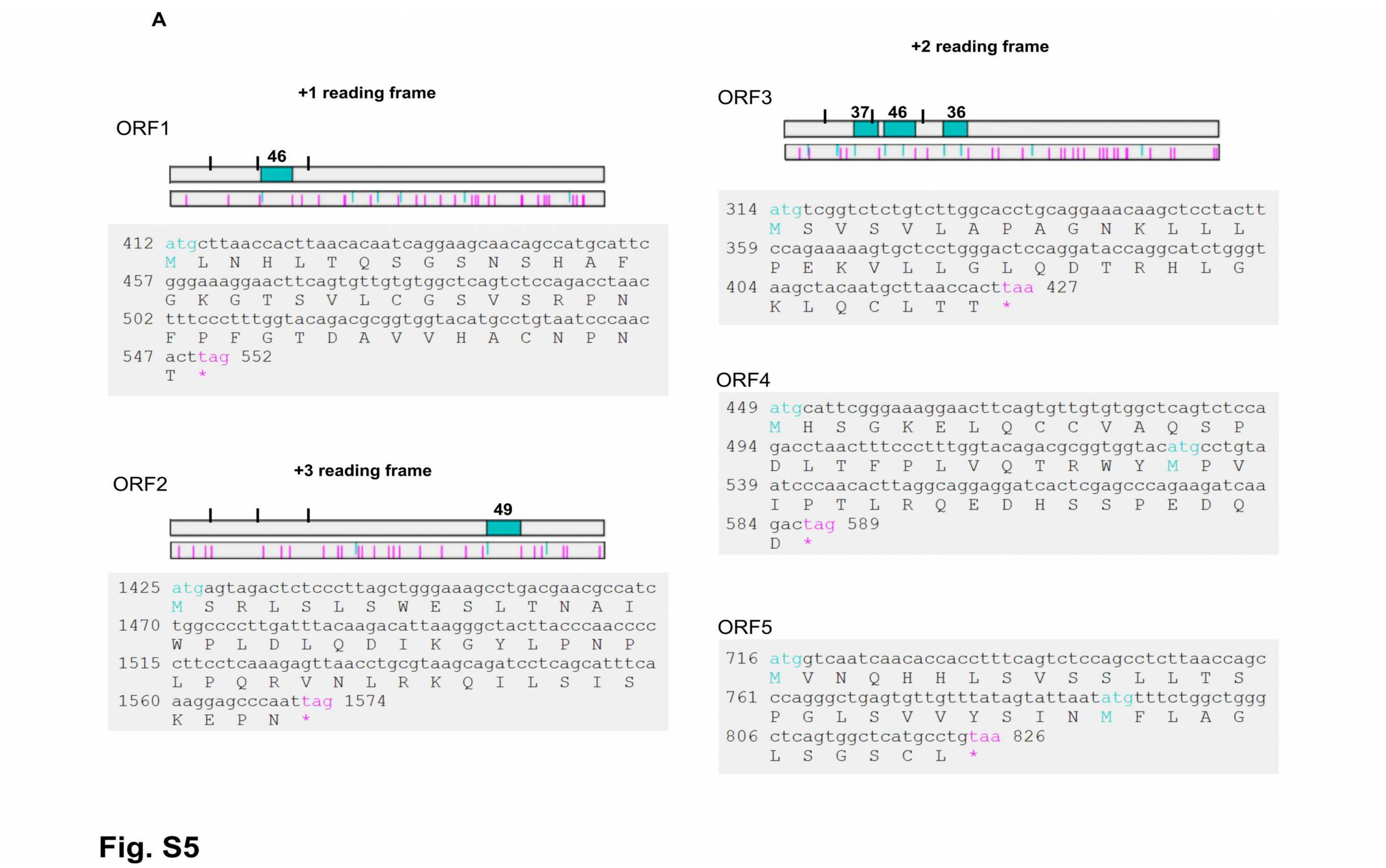

Fig. S5. Analysis of the sequence of BISPR for potential ORFs. The three reading frames and the potential ORFs longer than 30 amino acids are shown. The black, short vertical lines on top of the upper rectangle mark the location of exon-exon junctions. The numbers above each rectangle indicates the size of ORFs in amino acids. In the bottom rectangle the position of start and stop codons are shown (turquoise and purple, respectively).

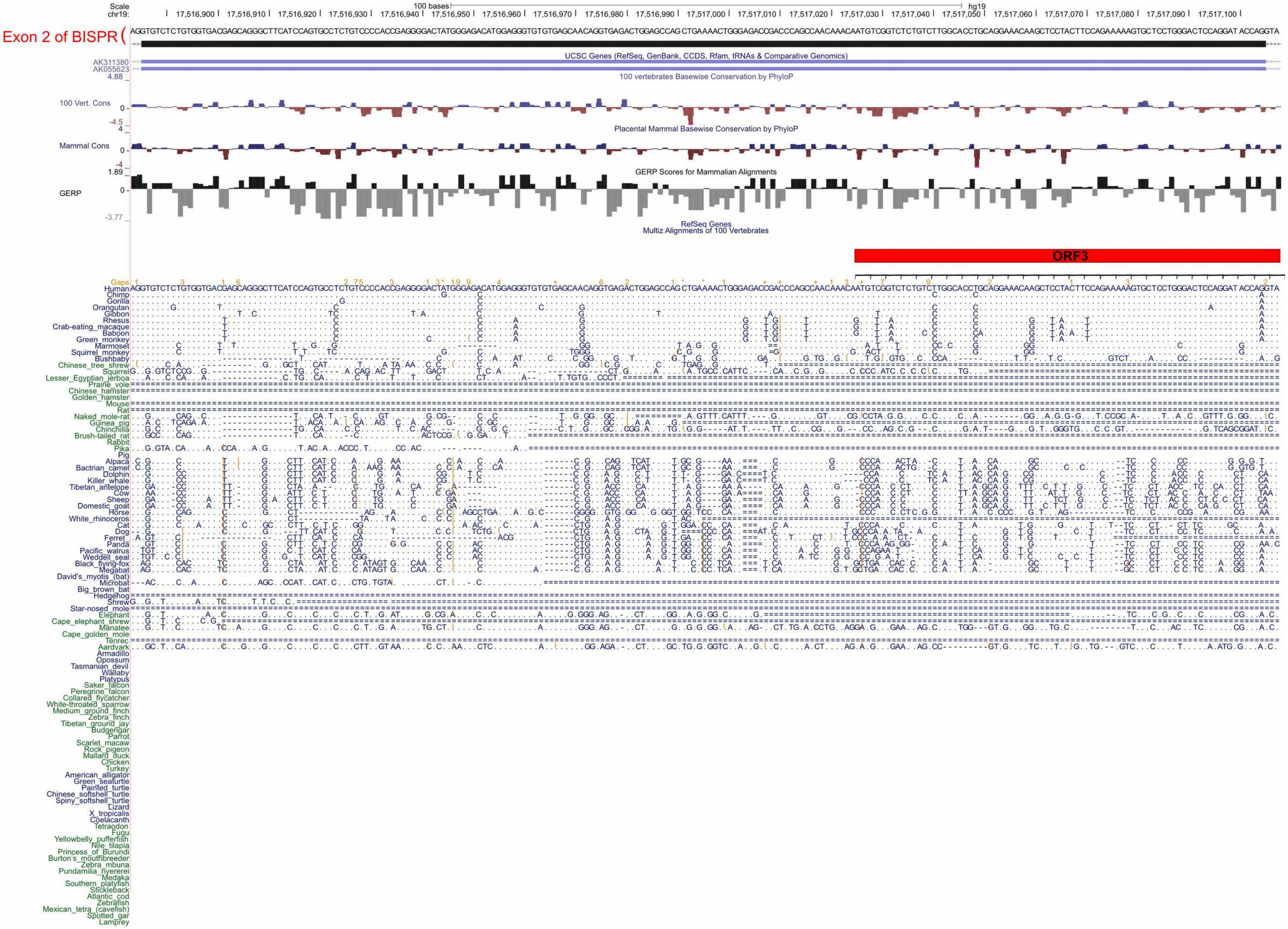

Scale
chr19:
100 bases
hg19
Exon 2 of BISPR
UCSC Genes (RefSeq, GenBank, CCDS, Rfam, tRNAs & Comparative Genomics)
AK311380
AK055623
100 vertebrates Basewise Conservation by PhyloP
100 Vert. Cons
Placental Mammal Basewise Conservation by PhyloP
Mammal Cons
GERP Scores for Mammalian Alignments
GERP
RefSeq Genes
Multiz Alignments of 100 Vertebrates
ORF3
Gaps
Human

Fig. S6A

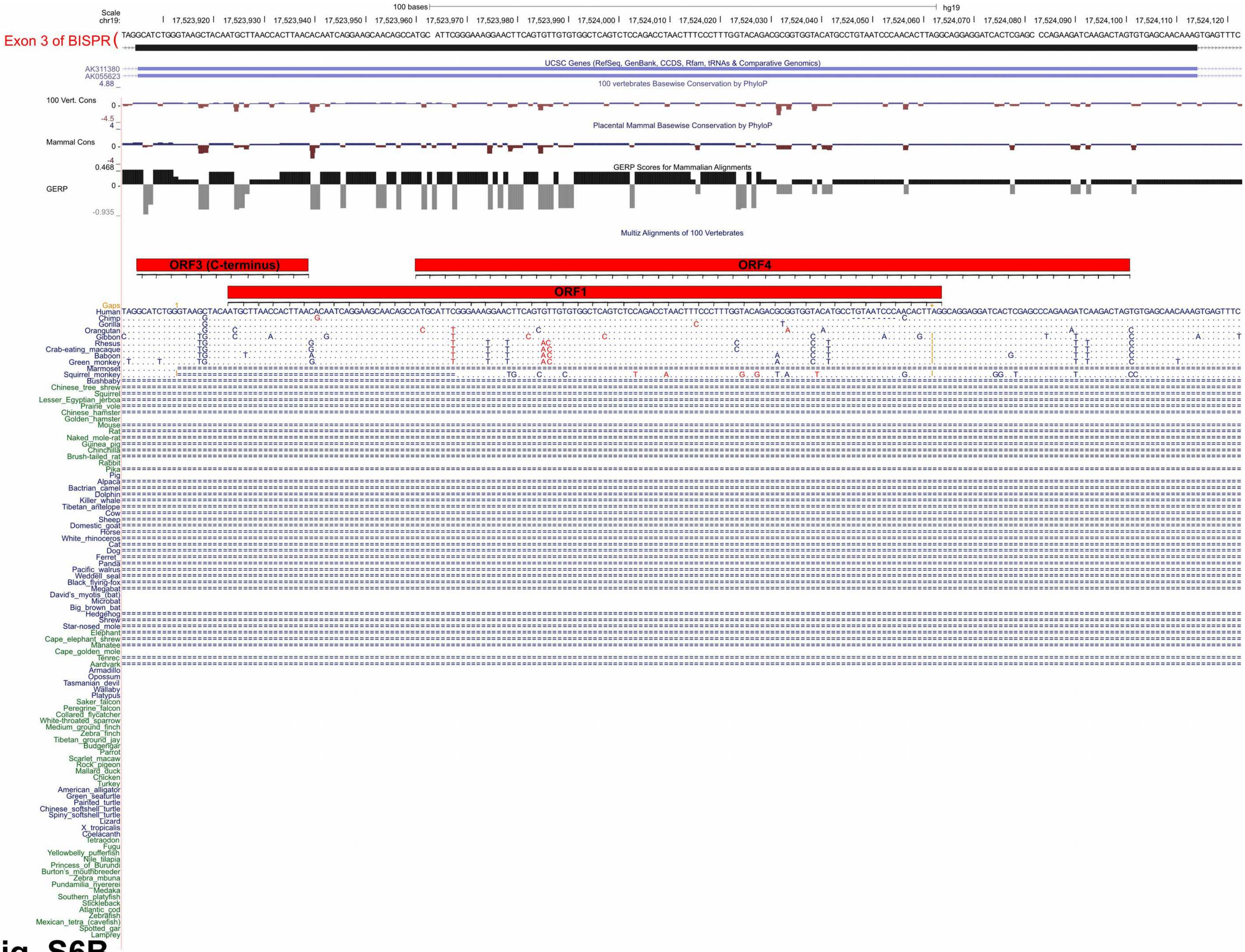

Exon 3 of BISPR
ORF3 (C-terminus)
ORF4
ORF1
UCSC Genes (RefSeq, GenBank, CCDS, Rfam, tRNAs & Comparative Genomics)
100 vertebrates Basewise Conservation by PhyloP
Placental Mammal Basewise Conservation by PhyloP
GERP Scores for Mammalian Alignments
Multiz Alignments of 100 Vertebrates

Fig. S6B

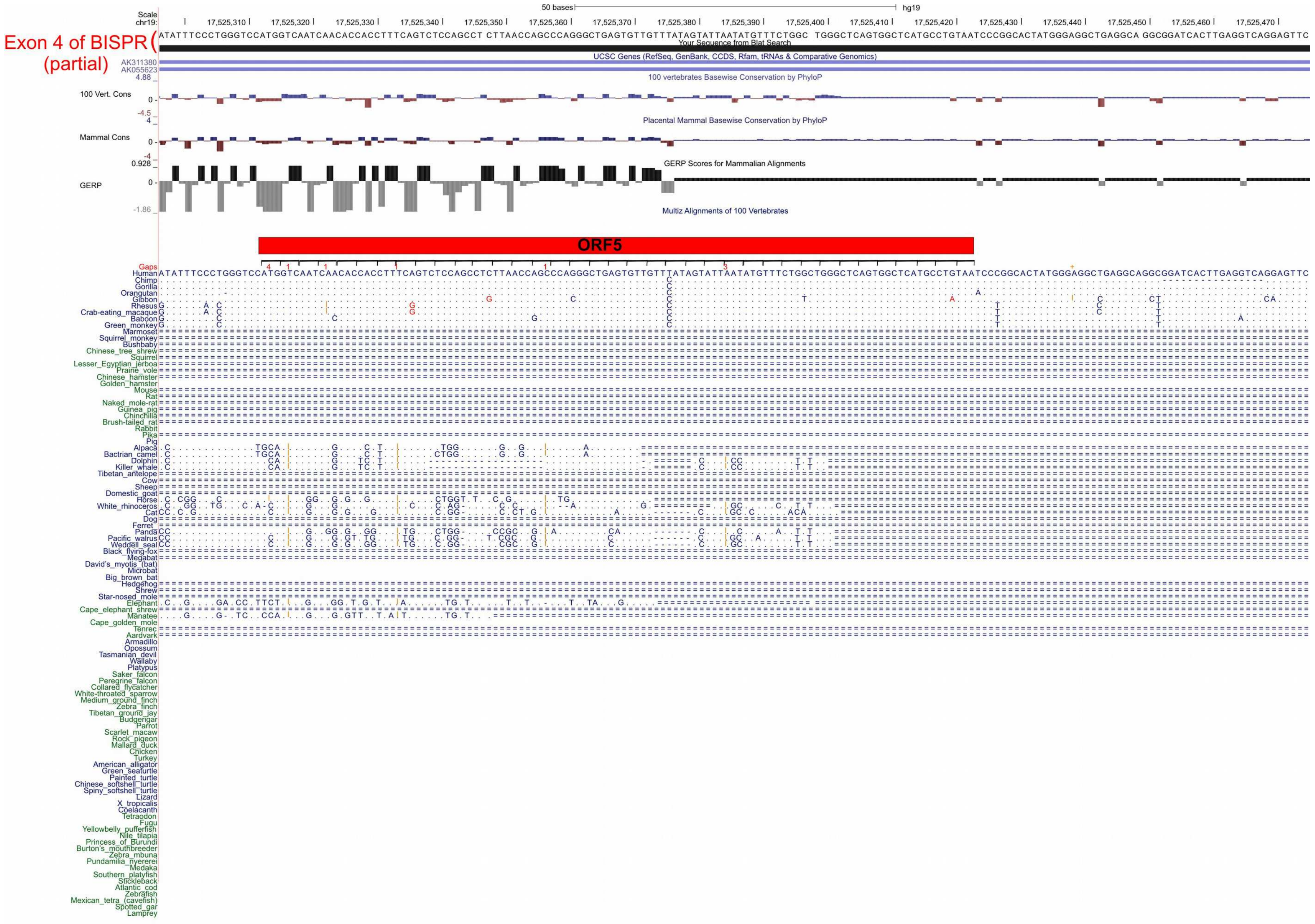

Exon 4 of BISPR (partial)
50 bases
hg19
Scale
chr19:
Your Sequence from Blat Search
AK311380
AK055623
UCSC Genes (RefSeq, GenBank, CCDS, Rfam, tRNAs & Comparative Genomics)
100 vertebrates Basewise Conservation by PhyloP
100 Vert. Cons
Placental Mammal Basewise Conservation by PhyloP
Mammal Cons
GERP Scores for Mammalian Alignments
GERP
Multiz Alignments of 100 Vertebrates
ORF5
Gaps

Fig. S6C

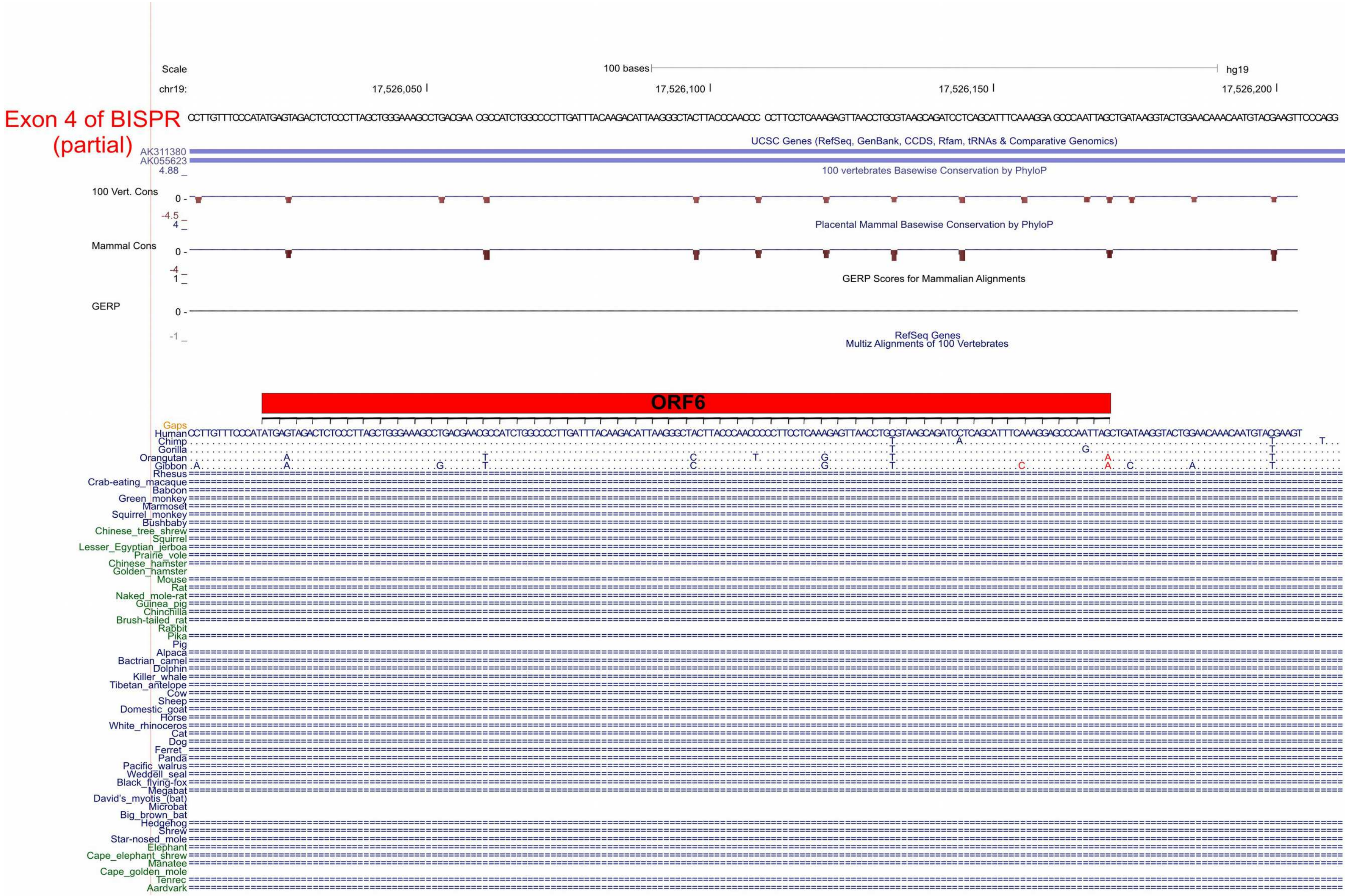
Exon 4 of BISPR (partial)
Scale
100 bases
hg19
chr19:
17,526,050
17,526,100
17,526,150
17,526,200
UCSC Genes (RefSeq, GenBank, CCDS, Rfam, tRNAs & Comparative Genomics)
AK311380
AK055623
100 vertebrates Basewise Conservation by PhyloP
100 Vert. Cons
Placental Mammal Basewise Conservation by PhyloP
Mammal Cons
GERP Scores for Mammalian Alignments
GERP
RefSeq Genes
Multiz Alignments of 100 Vertebrates
ORF6
Gaps
Human
Chimp
Gorilla
Orangutan
Gibbon
Rhesus
Crab-eating_macaque
Baboon
Green_monkey
Marmoset
Squirrel_monkey
Bushbaby
Chinese_tree_shrew
Squirrel
Lesser_Egyptian_jerboa
Prairie_vole
Chinese_hamster
Golden_hamster
Mouse
Rat
Naked_mole-rat
Guinea_pig
Chinchilla
Brush-tailed_rat
Rabbit
Pika
Pig
Alpaca
Bactrian_camel
Dolphin
Killer_whale
Tibetan_antelope
Cow
Sheep
Domestic_goat
Horse
White_rhinoceros
Cat
Dog
Ferret
Panda
Pacific_walrus
Weddell_seal
Black_flying-fox
Megabat
David's_myotis_(bat)
Microbat
Big_brown_bat
Hedgehog
Shrew
Star-nosed_mole
Elephant
Cape_elephant_shrew
Manatee
Cape_golden_mole
Tenrec
Aardvark

Fig. S6D

Fig. S6. The conservation pattern of potential ORFs in BISPR. The position in the chromosome and sequence of the RNA is shown on top. The specific region of the RNA being shown is indicated to the left, near the top in large red font. Three conservation tracks (100 vertebrates basewise conservation, placental mammal basewise conservation both by PhyloP, and GERP rejected substitution score for mammalians) are shown and their identity is indicated above each track and to the left. The ORF is shown in red, with the reading frame shown below it as a horizontal line with tick marks separating the codons. The number of ORFs correspond to those in Fig. S5. The unchanged sequences are shown as dots. Changed sequences shown in red preserve the codon identity, while those in blue lead to non-synonymous change. The orange/red numbers immediately above the aligned sequences indicate the presence of insertions or deletions at the corresponding position, with the number of inserted/deleted nucleotides shown. Overall, the predicted ORFS have an unfavorable ratio of synonymous to non-synonymous codon changes induced as a result of phylogenetic sequence variations even across very short evolutionary distances. For example, for ORFs 1, 5 and 6, the ratio of synonymous/non-synonymous changes are 14/21, 3/5 and 2/9, respectively.
